# A Facile Method of Preparing the Asymmetric Supercapacitor with Two Electrodes Assembled on a Sheet of Filter Paper

**DOI:** 10.3390/nano9091338

**Published:** 2019-09-19

**Authors:** Shasha Jiao, Tiehu Li, Chuanyin Xiong, Chen Tang, Alei Dang, Hao Li, Tingkai Zhao

**Affiliations:** 1 School of Materials Science and Engineering, Northwestern Polytechnical University, Xi’an 710072, China; usershasha@sina.com (S.J.); mecdoll@yahoo.com (C.T.); dangalei@nwpu.edu.cn (A.D.); lihao@nwpu.edu.cn (H.L.); ztk@nwpu.edu.cn (T.Z.); 2 School of Materials Science and Engineering, Shaanxi University of Science and Technology, Xi’an 710072, China

**Keywords:** carbon nanotubes, metal oxide, zinc oxide, ferroferric oxide, asymmetric supercapacitor

## Abstract

An asymmetric supercapacitor was prepared on a sheet of filter paper with two modified surfaces acting as electrodes in 1 M potassium hydroxide aqueous solution. By choosing carbon nanotubes and two different kinds of metal oxides (zinc oxide and ferro ferric oxide) as electrode materials, the asymmetric supercapacitor was successfully fabricated. The results showed that this device exhibited a wide potential window of 1.8 V and significantly improved electrochemical performances of its counterparts. Particularly, the one-sheet asymmetric supercapacitor demonstrated high energy density of 116.11 W h/kg and power density 27.48 kW/kg, which was attributed to the combined action and shortened distance between the two electrodes, respectively. Besides, it showed superior electrochemical cycling stability with 87.1% capacitance retention under room temperature. These outstanding results can not only give researchers new insights into compact energy storage systems, but they also provide a good prospect for flexible asymmetric supercapacitors.

## 1. Introduction

With the exhaustion of fossil fuels and the growing emphasis on environmental pollution problems, in recent years, it has become urgent to find a perfect source of alternative energy. Supercapacitors, which help bridge the gap between conventional capacitors and batteries in energy and power densities, have been drawing much attention due to the excellent performances and various promising potential applications [1,2,3,4]. Naturally, large supercapacitors are often used for backup power sources of regenerative hybrid electric vehicles because of their quick charging and long cycling life. Moreover, the fast development of intelligent and smart materials have contributed significantly to the application of supercapacitors in various fields, such as medical treatment, wearable technology, military devices and so on [5,6,7,8,9]. Generally, according to the different energy storage mechanisms, supercapacitors can be divided into two groups: Electrical double layer capacitance (EDLC) and pseudo-capacitance [10,11,12]. For the former, energy storage depends on the adsorbed ion by electrostatic charge between the electrode/electrolyte interface. However, the pseudo-capacitance is based on the redox reaction of active materials on the electrode surface. There are three types of electrode materials for supercapacitors, which mainly include high surface area activated carbons, transition metal oxides and conductive polymers [13,14,15,16]. Currently, in order to improve energy density and power density, hybrid supercapacitor devices have been widely studied by researchers from home and abroad, for carrying both the merits of EDLC and pseudo-capacitance [17,18,19]. In addition, according to the formula of energy density for supercapacitors, E = $\;\frac{1}{2}\;$CV^2^, it can be deduced that energy density is determined by specific capacitance (C) and the potential window (V). With large capacitance and potential window, it is clear to see that the energy density of the supercapacitor will definitely grow. Therefore, the preparation of asymmetric supercapacitors (AS) is also a promising approach to improve the electrochemical properties greatly in the same electrolyte [20,21,22]. The reason for this is that the AS can effectively increase the maximum operation voltage benefited from the different potential windows of electrodes, thus resulting in an increased specific capacitance and a high energy density.

Compared with the conventional carbon-based materials, carbon nanotubes (CNTs) show some special properties such as high surface area, strong absorbability and good electrical conductivity, which have been widely used in the research of EDLCs. Recently, many efforts have been devoted to the progress of CNTs in hybrid supercapacitor applications [15,23]. To sum up, the common types of CNT based composite electrode materials can be roughly divided into the following three categories: CNTs/metal composites, CNTs/metal oxide composites and CNTs/conductive polymer composites, respectively [11,23,24,25,26].

Zinc oxide (ZnO) is a kind of typical semiconductor with interesting piezoelectric, optoelectronic, and pyroelectric properties, which has been used extensively for optoelectronic devices, solid state gas sensors, production of green-blue ultraviolet, light emitting diodes and thin planar waveguides [27,28,29]. Recently, literature related to the suitability of ZnO as a potential candidate for supercapacitor has been gradually reported. Lo et al. successfully synthesized the ZnO/Ni(OH)_2_ nanocomposite under optimal pulse electrodeposition conditions. The results exhibited a large specific capacitance of 1830 F/g, a high energy density of 51.5 W h/kg, and a high power density of 9 kW/kg, respectively [30]. In view of the low cost, facile preparation, environmental friendliness and other unique characteristics, ferroferric oxide (Fe_3_O_4_) was extensively applied in many fields, such as microwave absorption, catalyst carrier, energy storage, sensing agent, magnetic recording and drug loading/delivery [31,32], etc. So far there is a growing concern over the possibility of Fe_3_O_4_ as electrode materials for supercapacitors and batteries due to the high theoretical capacity of its excellent electrochemical properties. Xia et al. reported high-quality mesoporous Fe_3_O_4_ nanocages as anode material for rechargeable lithium-ion batteries prepared by a complex-coprecipitation methods [33]. The unique anode demonstrated high specific capacitance and superior cycling performances at high and low current rates. Sun et al. fabricated a Fe_3_O_4_/Fe-CNTs nanocomposite as electrode materials for supercapacitors at room temperature with a new chemical synthesis route. The as-prepared Fe_3_O_4_/Fe-CNTs composite displayed a high specific capacitance of 1065 F/g at the current density of 1 A/g, which maintained 88% of the initial value after 1000 cycles [34].

In recent years, flexible electronics have attracted widespread attention with the development of smart materials. It not only has excellent mechanical properties but also enables them to be bended easily and mounted on various surfaces. Therefore, it is vital to explore substrate materials for batteries and supercapacitors with high flexibility. Among a long list of flexible substrate candidates, filter paper can serve as both flexible substrate and a separator of the supercapacitor due to the notable properties of its ability to bend without breaking and its particular network structure of sufficient porosity to let the electrolyte pass through totally between two electrodes [35,36,37,38,39].

Herein, this paper developed a novel AS with two electrodes assembled on a sheet of filter paper only by a kind of simpler method. Through designing an asymmetric device, the potential window of the AS was enlarged. The energy and power densities increased accordingly. In addition, this study also discussed the composition, microstructure, morphological characteristics and electrochemical properties of the as-synthesized electrode and AS materials. The results provided an innovative idea and unique technique for fabricating high performances of AS. Furthermore, the achievements will lay a foundation for the development and improvement of flexible energy storage system.

## 2. Materials and Methods

### 2.1. Materials

CNTs were procured by Beijing Chemical Works, Beijing, China and the purity was greater than 95%. In order to get more functional groups, the raw CNTs were pre-acidated by strong acid treatment. Filter paper (FP), potassium hydroxide (KOH), 98% concentrated sulfuric acid (H_2_SO_4_) and 68% concentrated nitric acid (HNO_3_) were purchased from Xi’an Sanpu Fine Chemical Factory, Xi’an, China. Further, Fe_3_O_4_ and ZnO were purchased Sinopharm Chemical Reagent Co. Ltd., China. All materials were commercially available and used as received.

### 2.2. Preparation of Flexible AS on One-Sheet of FP

**Figure 1:**
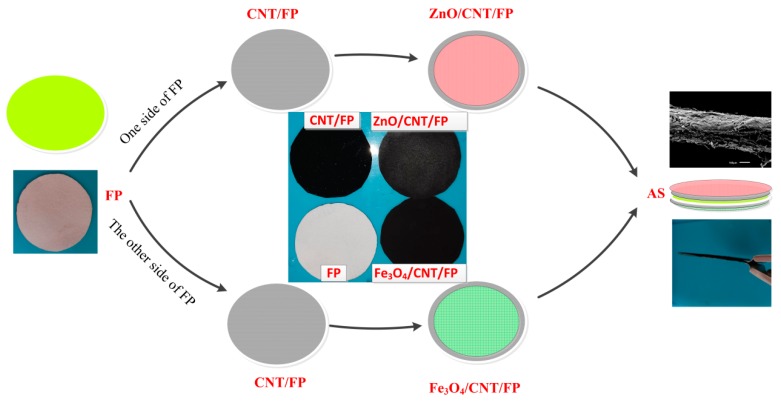
Schematic diagram of the preparing process of zinc oxide (ZnO)/carbon nanotubes (CNT)/filter paper (FP), Fe_3_O_4_/CNT/FP and asymmetric supercapacitors (AS) on one sheet of FP, respectively.

Firstly, the raw CNTs underwent a treatment with concentrated H_2_SO_4_ and HNO_3_ to improve their suspension in water. Subsequently, 20 mg of CNTs were added in 50 mL deionized water by stirring and ultrasonic dispersing to obtain highly water-dispersible and an equably distributed CNT mixture. This method works the same for preparing 50 mL ZnO suspension (3 mg/mL). Following that, based on the vacuum filtration technology, CNTs and ZnO were successively deposited on FP through controlling filtration suction time and pressure. After being dried in the air, the flexible ZnO/CNT/FP electrode was successfully fabricated. The procedures for preparation of Fe_3_O_4_/CNT/FP film was similar to that of ZnO/CNT/FP except for the suspension of Fe_3_O_4_ (2 mg/mL). Finally, the FP was coated with CNTs and ZnO on one side, while CNTs and Fe_3_O_4_ were deposited on the other side. Five samples of AS were prepared to investigate performance. Figure 1 illustrates the schematic diagram of the preparation process.

It is critically important to evaluate the mass of active materials deposited on each side of FP. Based on the charge balance strategy ${\;Q}_{+}=Q_{-}$, the optimal mass ratio was calculated by the following equations below:

(1) $$Q_{+}=C_{+}•\Delta E_{+}•m_{+}$$

(2) $$Q_{-}=C_{-}•\Delta E_{-}•m_{-}$$

where $Q_{+}$ and $Q_{-}$ are the stored charges on the positive and negative electrodes, respectively. ∆E_+_ and ∆E_−_ relate to the potential ranges of each electrode. Where $C_{+}$ and $C_{-}\;$ are the specific capacitance of the positive and negative electrodes, respectively. $m_{+}$ and $m_{-}$ are the mass of electroactive materials on the electrodes [40,41]. Therefore, the optimal mass ratio ($m_{+}:m_{-}$) is obtained by the formula below:

(3) $$\frac{m_{+}}{m_{-}}=\frac{C_{-}•\Delta E_{-}}{C_{+}•\Delta E_{+}}\;$$

Combined with the equation for calculating the specific capacitance (C_m_) below, the optimal mass ratio of positive electrode to negative one was 0.3125:

(4) $$C_{m}=\frac{1}{m•\gamma •\Delta V}\int_{V_{0}}^{V_{0}+\Delta V}\mathrm{IdV}\;$$

where m is the mass of active materials, γ represents the scanning rate, I indicates the current response, ΔV is the operating potential window, and V is the voltage, respectively. Besides, the corresponding energy density (E) and power density (P) can be obtained by the following formulas [42]:

(5) $$E=\frac{C_{m}•V^{2}}{2}\;$$

(6) $$P=\frac{E}{t}\;$$

where C_m_ is the specific capacitance, V denotes the potential window and t stands for the discharging time.

### 2.3. Characterization and Electrochemical Measurements

The chemical compositions and morphology of the samples were examined by X-ray diffraction (XRD; Cu-Kα λ = 1.541 Å, 2θ = 10~80°), Raman spectroscope (Raman spectrometer Labram ARAMIS, HR800, Jobin Yvon LabRam, Japan ), field-emission scanning electron microscopy (FE-SEM; Hitachi S-5200, Japan), and high-resolution electron scanning microscopy (HRTEM, JEM2100, JEOL, Japan). An electrochemical workstation (CHI 660D) was employed to test the electrochemical performances of the composites by using 1 M KOH solution as electrolyte at room temperature.

## 3. Results and Discussion

### 3.1. Structure and Morphology

**Figure 2:**
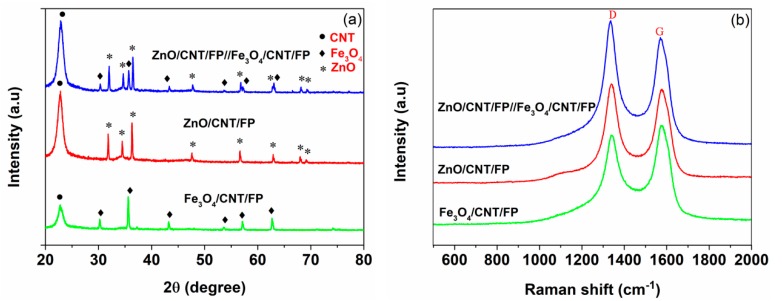
(**a**) XRD patterns of ZnO/CNT/FP, Fe_3_O_4_/CNT/FP and ZnO/CNT/FP/Fe_3_O_4_/CNT/FP composites, respectively; (**b**) Raman spectra of ZnO/CNT/FP, Fe_3_O_4_/CNT/FP and ZnO/CNT/FP//Fe_3_O_4_/CNT/FP composites, respectively.

The XRD patterns of ZnO/CNT/FP, Fe_3_O_4_/CNT/FP and ZnO/CNT/FP//Fe_3_O_4_/CNT/FP composites are presented in Figure 2a. Typically, for CNT/Fe_3_O_4_/FP, there were diffraction peaks around 30.3°, 35.7°, 43.3°, 54.1°, 57.4° and 62.9°, well corresponding to the diffraction characteristics of cubic Fe_3_O_4_ [43]. However, diffraction peaks located at 2θ around 31.6°, 34.5°, 36.2°, 47.4°, 56.5°, 62.8°, 68.1° and 69.3° of ZnO/CNT/FP hybrid suggested (100), (001), (101), (102), (110), (103) (112) and (201) lattice planes of ZnO, respectively [44,45,46]. Furthermore, the composites deposited on both sides of the same FP were also observed. A strong peak was clearly observed from the XRD curve of ZnO/CNT/FP//Fe_3_O_4_/CNT/FP hybrid, which was the same case with ZnO/CNT/FP and Fe_3_O_4_/CNT/FP electrodes. The reason for this was attributed to the diffraction characteristics of CNT. The XRD results of ZnO/CNT/FP//Fe_3_O_4_/CNT/FP showed that the distinct peaks of CNT, ZnO and Fe_3_O_4_ were found on the intensity curve of one sheet FP composite. The structural characteristics of the as-prepared electrodes and AS were further determined by Raman spectra. As illustrated in Figure 2b, two typical Raman peaks existed in the Raman spectroscopy, indicating D and G bands of CNT in the composites of the test samples, which approved the results of the above-mentioned XRD analysis.

**Figure 3:**
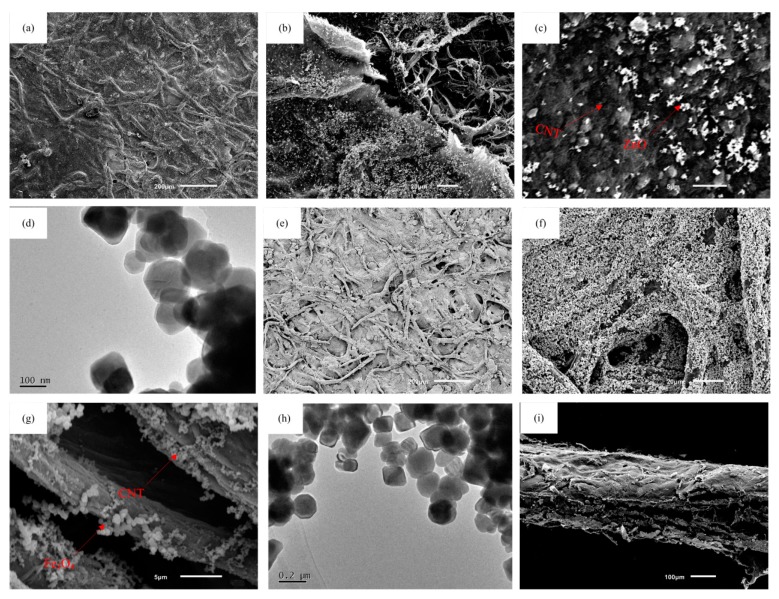
(**a**) Low power of the surface morphology image of the ZnO/CNT/FP electrode; (**b**) SEM image of the cellulose fiber of the ZnO/CNT/FP electrode; (**c**) high power image of the ZnO/CNT/FP electrode; (**d**) TEM image of the ZnO/CNT/FP electrode; (**e**) low power of the surface morphology image of the Fe_3_O_4_/CNT/FP electrode; (**f**) SEM image of the cellulose fiber of the Fe_3_O_4_/CNT/FP electrode; (**g**) high power image of the Fe_3_O_4_/CNT/FP electrode; (**h**) TEM image of the Fe_3_O_4_/CNT/FP electrode; (**i**) section morphology images of the AS.

The surface and section morphology images of AS are displayed in Figure 3. Figure 3a–d display the micrographs of the ZnO/CNT/FP composite. As illustrated in Figure 3a,b, the fiber skeleton of FP was uniformly deposited with a solid and continuous layer of CNTs and grain growth of ZnO, which can improve the electrochemical activity of the FP substrate. At high magnification, microstructure investigation of Figure 3c clearly showed that the white ZnO nanocrystals were anchored onto a nest-like pattern of CNTs with three dimensional porous FP as a support, consisting with the XRD and Raman results. Figure 3d displayed the ZnO nanoparticles by the HRTEM image. Furthermore, the surface structures of the Fe_3_O_4_/CNT/FP composite electrode were also obtained by SEM. Figure 3e,f displays the representative images of Fe_3_O_4_/CNT/FP film under different magnifications. As shown in Figure 3e, the surface of the three-dimensional cellulose FP was covered with a dense and continuous layer of substances. It was illustrated clearly in Figure 3f,g that the fine particles of Fe_3_O_4_ were uniformly distributed throughout the interior and surface of the FP. In addition, further observation was performed to reveal the details about the surface of the Fe_3_O_4_/CNT/FP film. Figure 3h displays the cubic structure of Fe_3_O_4_ and entangled tube-shaped CNT, making the electrode a better electrochemical activation. Further, the fracture surface of AS studied by SEM images in Figure 3i shows a 3D porous structure sandwiched between two electrode materials, which made it a unitized configuration of electrodes and separator assembled on a piece of FP. I indicated that the average thickness of the as-synthesized AS sheet was around 150 μm; this being much thinner and lighter than most supercapacitors.

### 3.2. Electrochemical Tests of the As-Prepared Electrodes and AS

**Figure 4:**
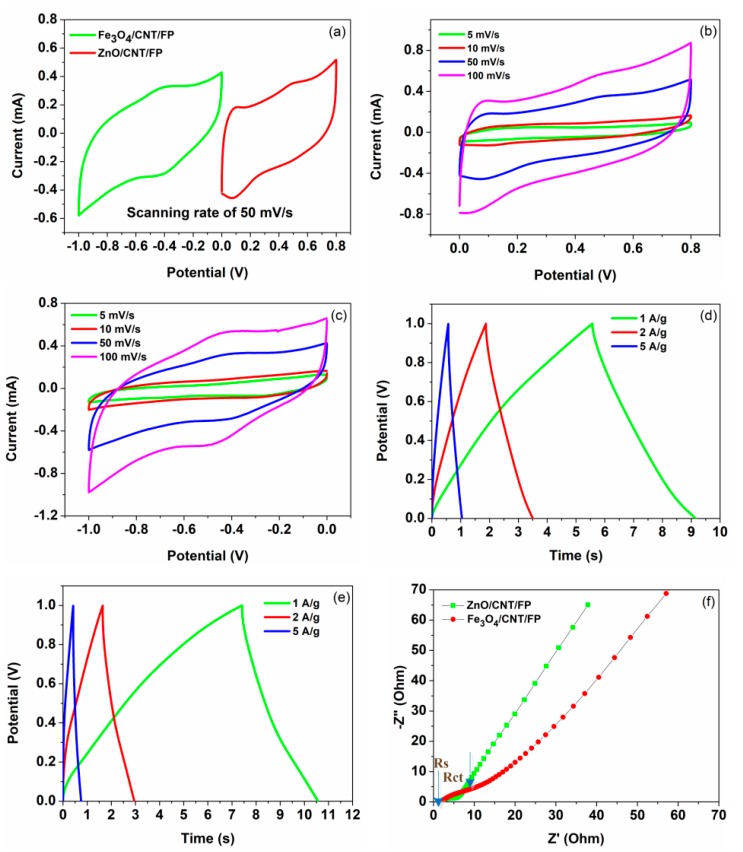
(**a**) Cyclic voltammetry (CV) curves of ZnO/CNT/FP and Fe_3_O_4_/CNT/FP electrodes at the scanning rate of 50 mV/s; (**b**) CV curves of ZnO/CNT/FP at different scanning rates; (**c**) CV curves of Fe_3_O_4_/CNT/FP at different scanning rates; (**d**) galvanostatic charge/discharge (GCD) curves of the ZnO/CNT/FP electrode at different current densities; (**e**) GCD curves of the Fe_3_O_4_/CNT/FP electrode at different current densities; (**f**) Nyquist plots of the ZnO/CNT/FP and Fe_3_O_4_/CNT/FP electrodes, respectively.

The as-prepared ZnO/CNT/FP and Fe_3_O_4_/CNT/FP films were systematically investigated in an aqueous solution of KOH (1 M). A conventional three-electrode system with each sample as a working electrode, (Ag/AgCl as a reference electrode and platinum plate as a counter electrode), were applied to measure the electrochemical performances, respectively. A comparison of the potential range determined by cyclic voltammetry (CV) results in Figure 4a indicated the optimized stable potential window of ZnO/CNT/FP was 0–0.8 V (vs. Ag/AgCl), while that of Fe_3_O_4_/CNT/FP was −1.0–0 V (vs. Ag/AgCl) under the same scanning rate of 50 mV/s. According to experimental results of the CV test under different scan rates, the CV curves of ZnO/CNT/FP nanosheet demonstrated in Figure 4b had a pair of symmetrical and steady peaks and a relatively closed area, revealing the large peak current values, which showed that the electrochemical reversibility and activity of ZnO/CNT/FP were good. These results were attributed to the electrochemically active layer of nanocrystalline ZnO and nano-scale particles of CNT of the CNT/ZnO/FP electrode, which enhanced the specific capacitance through a combination of metal oxide and carbon nanomaterials with the electrolyte. Meanwhile, as shown in Figure 4c, the shape of the CV curves of Fe_3_O_4_/CNT/FP revealed that the capacitance characteristics were different from that of CNTs, the shape of which was typically an ideal rectangle, thereby indicating that Fe_3_O_4_ nanoparticles mainly provided the pseudo-capacitance effect on the composite electrode material. It was also noted that the current reactions changed along with the increase of scanning rates, but this was not exactly linear. Galvanostatic charge/discharge (GCD) tests of ZnO/CNT/FP and Fe_3_O_4_/CNT/FP composite electrodes were conducted in 1 M KOH (aq) at various current densities of 1, 2 and 5 A/g, respectively. The plots of voltage versus time corresponding to the results of the two electrodes are shown in Figure 4d,e. It can be concluded from these plots that the discharge time increased with the decrease of current density. Even if the minimum current density was 1 A/g, the charge and discharge time in all of the prepared films was only around 10 s. Moreover, the relationships between the potential and the charge or discharge time was not accurately linear in the GCD curves. There were a few minor deviations from a straight line, which indicated the pseudocapacitive behaviors of the electrode material in the energy storage mechanism. Besides, because of the resistance of the electrode materials, the charge time of the sample was a little bit longer than the discharge time during cycling. On the other hand, the charging and discharging curves were nearly symmetric, demonstrating a reversible electrochemical behavior resulted from the introduction of CNT, which had more comparative advantages of the double layer capacitor effect. Moreover, electrochemical impedance spectroscopy measurements were also carried out to further illustrate the performances of electrode nanocomposites. It is displayed in Figure 4f that the resistance of electrode-electrolyte interfaces (R_s_) of the two electrodes were 2.5 and 2.6 Ω, respectively, which were determined by the high-frequency intercept on the *X*-axis. The diameter of the semicircle in the lower-left corner of Figure 4f represented the charge transfer resistance of electrode (R_ct_), while the steep straight line at the low frequency showed an excellent ion diffusion behavior in the electrolyte. According to the figures given below, it was obvious that each electrode exhibited a certain similarity of lower R_s_ and R_ct_ values, indicating a competitive candidate in terms of high capacity and good cycle stability.

**Figure 5:**
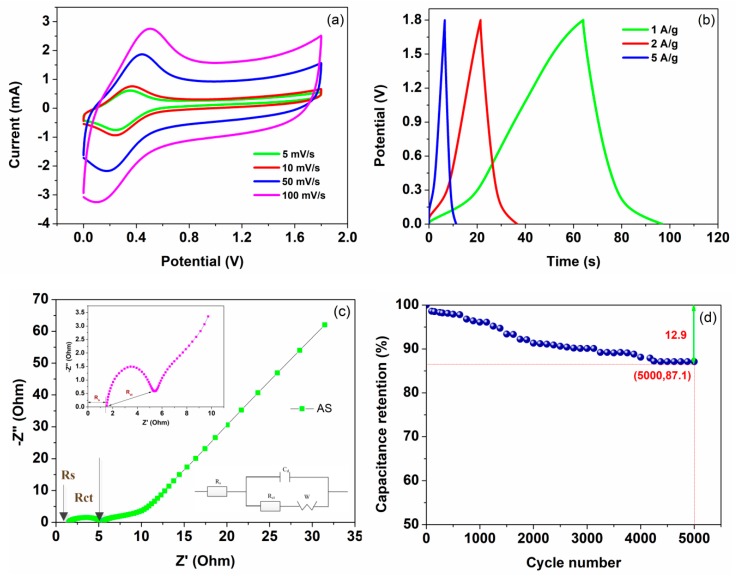
(**a**) CV curves of the AS at different scanning rates. (**b**) GCD curves of the AS at different current densities. (**c**) Nyquist plots of the AS. (**d**) Stability study for the AS.

**Figure 6:**
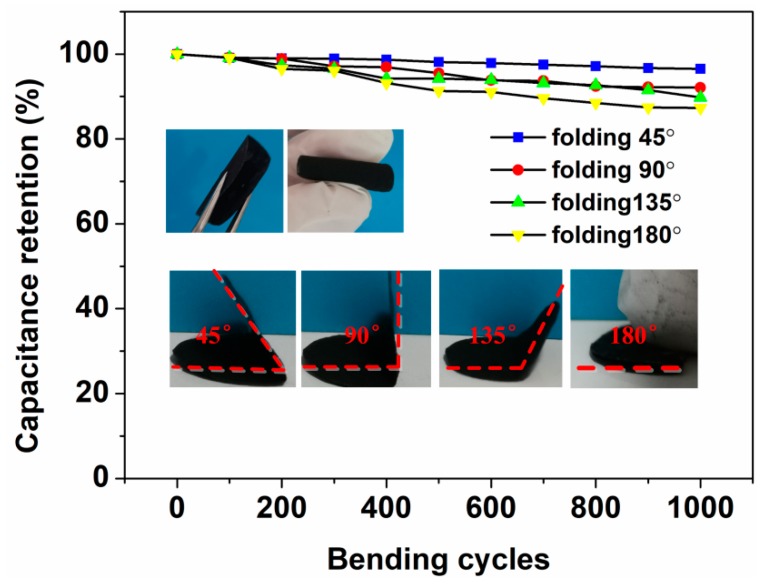
Capacitance retention of the AS at different folding degrees.

As displayed in Figure 5a, the CV curves of ZnO/CNT/FP/Fe_3_O_4_/CNT/FP AS operated at different scanning speeds ranging from 5 to 100 mV/s with the potential window of 1.8 V. The shape of the CV curves were well maintained without observation of any deformation even at the highest speed case of 100 mV/s, suggesting the outstanding rate capability of the device. Moreover, it can be seen from the curves of Figure 5a that a couple of suspected redox peaks around 0.2 V and 0.4 V were also observed and shifted positively with the increasing scanning speed. Obviously, the relationship between the current response and potential response was not linear, which was totally different from the electric double layer capacitors. This fully indicated by the faradic pseudo-capacitance of the AS. In addition, the CV curves demonstrated a typical characteristic of hybrid AS at different scanning rates, which was the result of combining the actions of EDLC and faradic behaviors. Subsequently, its electrochemical properties were evaluated by GCD measurements at various current densities of 1, 2 and 5 A/g, respectively. It is shown in Figure 5b that the typical triangular shape of GCD curves indicated an approximate linear relationship of voltage versus time, demonstrating excellent capacitive behavior in agreement with the results from the CV test above. Besides, the specific capacitances, energy densities and power densities of AS device were calculated at the current densities of 1, 2 and 5 A/g based on the relevant formulas and GCD curves, respectively. The calculated value of C_m_, E and P were listed and described in Table 1. The most significant improvement was that the ZnO/CNT/FP/Fe_3_O_4_/CNT/FP AS which exhibited remarkable energy/power densities which were superior to some of their normal types of counterparts. The first reason was the presence of the asymmetric configuration of the supercapacitor. Besides, the synergetic effect of electrode materials with nano/micro hierarchical structures (CNT, ZnO and Fe_3_O_4_) effectively increased the high rate capability and provided a larger surface area and short distance for ion diffusion, as well as electron transport. To investigate the resistance behaviors of the AS, Figure 5c displays the Nyquist plots fitted with Zview software by using an equivalent circuit model and the enlarged drawing of R_s_ and R_ct_. Obviously, the Nyquist plot showed a steep incline at low frequencies, which exhibited the good characteristics of the AS and small ion diffusion resistance in the electrolyte. As displayed in the inset image of Figure 5c, the intercept of the trend curve on the horizontal coordinate was the value of the equivalent series resistance (R_s_) of AS, which was about 1.4 Ω. Particularly, the arc-shaped curve of the enlarged image at high frequency area represented the value of R_ct_ and the typical pseudo-capacitive performance of AS. Finally, the electrochemical stability of the AS device was also tested by a long cycling life experiment at the current density of 2 A/g up to 5000 times. As shown in Figure 5d, the one-sheet ZnO/CNT/FP/Fe_3_O_4_/CNT/FP AS device demonstrated a remarkable long-term cycling stability with 12.9% deterioration of the original specific capacitance value after 5000 times of a charge–discharge test, which can be ascribed to the mass loss of the as-prepared ZnO/CNT/FP/Fe_3_O_4_/CNT/FP materials in the electrolyte during the charge–discharge process. For flexibility, Figure 6 displays the specific capacitance retention of the circular AS (2 cm in diameter) changing with folding times under different folding degrees of 45°, 90°, 135°and 180°, respectively. Remarkably, the calculated specific capacitance retention rate still remained on a high level (87.3%) even at the most frequently folding times of 1000 and maximum folding angle of 180°, respectively.

**Figure 7:**
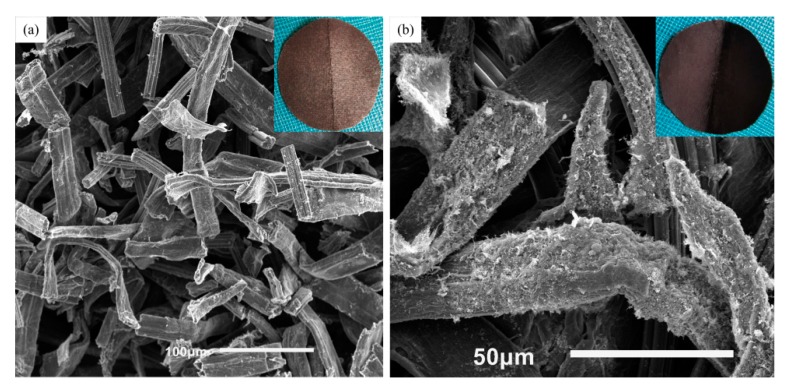
(**a**) SEM image of the AS electrode (ZnO/CNT/FP) after bending. (**b**) SEM image of the AS electrode (Fe_3_O_4_/CNT/FP) after folding.

**Figure 8:**
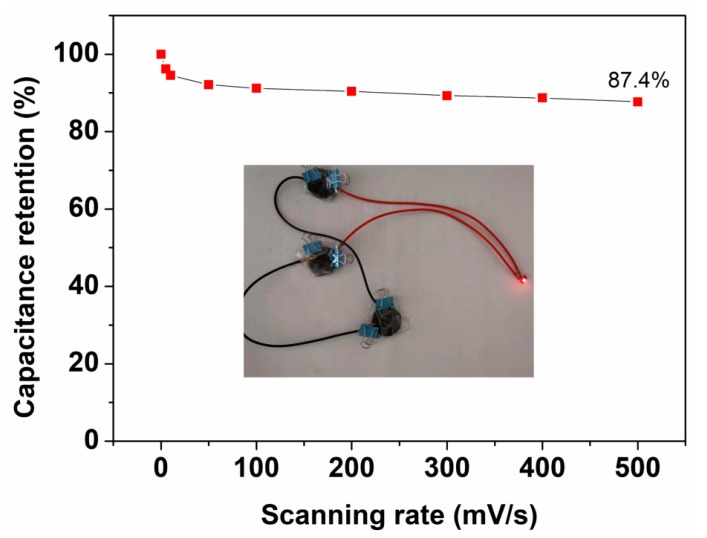
Rate stability of the AS and the application of powering the LED light (40 mW).

Figure 7 below shows the digital and SEM images of the AS electrodes after bending. As it can be seen from the photographs, there was a sharp crease with 2cm in length in the middle of the AS, which was equal to the diameter of the circular AS. To compare the changes of morphologies after folding, Figure 7a displayed the SEM image of the crack on the ZnO/CNT/FP electrode. After suffering from the repeated bending forces, the cellulose fibers of FP were destroyed and became discontinuous. But the electrochemical activities were still covered on the surface of each fiber, which prevented the rapid decline of the capacitance with the folding times increasing. However, the SEM image of cellulose fibers of the other electrode (Fe_3_O_4_/CNT/FP) in Figure 7b changed little after bending many times. The reason for this can be ascribed to the bending force acting directly on the electrode of ZnO/CNT/FP, resulting in the different force condition on the two sides of the AS. Figure 8 shows the curve of capacitance retention over the scanning rates. Remarkably, the capacitance retention of the as-prepared AS reached 87.4% even at the highest scanning rate of 500 mV/s. The excellent rate capability of the AS was attributed to the electrochemical activities on the surface and the reduced path of the electrolyte transport. A red LED light (40 mW) was lighted when it was connected to the fully charged power system (Figure 8 inset).

## 4. Conclusions

To summarize, a novel AS assembled on one-sheet of filter paper was synthesized by using a ZnO/CNT/FP nanocomposite as a positive electrode and Fe_3_O_4_/CNT/FP as a negative electrode, respectively. The comparative results clearly revealed that this smart and pocket-sized device demonstrated superior electrochemical performances (specific capacitance, energy density, power density and cycling stability) in potential applications for AS. On the other hand, this paper provided a new and effective method for manufacturing the integrated supercapacitor without special preparation of a separator. The conducting coatings of CNTs and pseudo-capacitance of metal oxides effectively covered the two surfaces of the filter paper, creating about 150 μm thick of interior layer, which was served as a separator of the one-sheet AS. These findings indicated that the as-prepared AS was considered to be a promising candidate as a high-performance, flexible electronic device. In addition, this kind of AS can be widely used in many fields, including energy storage and power output technologies for portable electronics, electric vehicles, and renewable energy systems operating on intermittent sources for the next generation.

## Figures and Tables

**Table 1:** The specific capacitance, energy density and power density of the AS at different current densities.

Current Density (A/g)	Specific Capacitance (F/g)	Energy Density (W h/kg)	Power Density (kW/kg)
1	139.12	125.21	13.74
2	129.00	116.11	27.48
5	100.19	90.17	68.70
